# Development of a new environmentally friendly and efficient centrifugal variable diameter metering device

**DOI:** 10.3389/fpls.2024.1404201

**Published:** 2024-07-03

**Authors:** Meng Zhang, Pengfei Zhao, Xiaojun Gao, Qinghui Lai

**Affiliations:** ^1^ Faculty of Mechanical and Electrical Engineering, Kunming University of Science and Technology, Kunming, China; ^2^ College of Mechanical and Electronic Engineering, Northwest A&F University, Yangling, China; ^3^ Education Ministry Key Laboratory of Renewable Energy Advanced Materials and Manufacturing Technology, Yunnan Normal University, Kunming, China

**Keywords:** seed metering device, centrifugal filling, pneumatic, response surface experiment, maize

## Abstract

**Introduction:**

The design of the maize metering device involves centrifugal variable diameter pneumatic and cleaning mechanisms, aiming to enhance the performance and power efficiency of pneumatic maize metering devices. Leveraging the impact of changes in centrifugal diameter and the guidance and positioning of airflow, we optimize the hole insert, seeding plate, seed limit board, and integrated front shell. This optimization facilitates the adjustment of both the quantity and posture of seed filling. As a result, seeds can form a uniform flow within the annular cavity, reducing the wind pressure necessary for regular operation and decreasing power consumption.

**Methods:**

A quadratic regression orthogonal rotation combination experiment is conducted using a self-made experiment bench, considering ground speed, wind pressure, and seeding rate as the experiment factors. Furthermore, a comparative experiment involving a novel centrifugal variable-diameter type metering device.

**Results:**

The results indicate optimal seeding performance when the ground speed is 13.2 km/h, the wind pressure is 1.2 kPa, and the feeding rate is 25 seeds/s. Under these conditions, the quality of feed index reaches 95.20%, the multi-index is 3.87%, and the miss index is 0.93%. Findings reveal that the developed seed metering device achieved a quality of feed index exceeding 93.00% across varying speeds of 12~18 km/h, aligning with the production requirements. Moreover, the actual power consumption of Type B and C is about 85.00% and 98.00% lower than Type A, standing at only 32.90 W at 18 km/h. The COP of Type C is about 86 times and 12 times that of Type A and B, respectively, meeting the demands for efficient production of maize seed metering devices.

**Discussion:**

In comparison to traditional design and structural parameter optimization methods for maize seed metering device, this study is helpful to the sustainable development of maize industry and reduce environmental pollution.

## 1 Introduction

Maize serves as a pivotal agricultural crop, contributing to animal feed, ethanol biofuels, and human consumption. Its significance lies in nourishing an expansive and burgeoning global population, albeit with the potential for pollution during production, impacting ecosystems and human health (Hill et al., 2019; Cisternas et al., 2020; Wei et al., 2023). Production methods are progressing towards greater efficiency and environmental cleanliness (Xiong et al., 2022). The primary objective of high-speed precision seeding technology is to ensure superior seeding quality while executing high-speed operations. The realization of this goal relies fundamentally on deploying a high-precision maize metering device (Li et al., 2021; Tang et al., 2022). Challenges such as population accumulation, squeezing, and friction among seeds impede seed-filling and substantially reduce seed-filling time, significantly impacting seed-filling performance. Moreover, the heightened velocity amplifies the centrifugal effect on seeds, exacerbating their instability within the seeding plate hole. Which is an influential factor constraining the swift evolution of precision seeding (Gao et al., 2022b). In response to the demands of efficient production, the structure of the maize metering device is progressively evolving into a more intricate form. However, this complexity increases work energy consumption. Regrettably, this trend is not aligned with the principles of clean and sustainable development in the maize industry (Bai et al., 2019; Zhu et al., 2022; Shah et al., 2023). Hence, the development of an efficient, sustainable, energy-saving, and emission-reducing seed-metering device emerges as pivotal for fostering the health of the maize industry.

Maize metering devices can be categorized into two types based on their operational principles: mechanical and pneumatic (Ren & Yi, 2022). The mechanical maize metering device relies solely on the seed’s gravity for filling and clearing. Achieving the necessary accuracy at high speeds becomes challenging, with an increased risk of seed damage and low energy efficiency. This inability to adapt to the sustainable, clean production requirements of high-speed precision seeding is evident (Lei et al., 2021a; Du and Liu, 2023; Sun et al., 2023). In contrast, the pneumatic maize metering device showcases robust adaptability to seeds (Sun et al., 2020; Lei et al., 2021b; Li et al., 2023b). For example, Wang et al. (2021) designed an inside-filling maize metering device, investigating the correlation between ground speed and working wind pressure. Results indicated excellent seeding performance when the ground speed was 13.10 km/h and the working wind pressure was 4.75 kPa. Tang et al. (2023) proposed a pneumatic type of maize metering device. Results indicated optimal parameters, including a ground speed of 10 km/h and a wind pressure of 3 kPa in the tube. Wang et al. (2023) analyzed the high-speed sowing performance of three maize varieties at ground speeds exceeding 12 km/h. The results revealed the lowest miss index at negative pressures exceeding 6 kPa. Zhao et al. (2024) used numerical simulation and high-speed camera were employed to investigate the impact of diversion turbine structure, dynamic wind pressure, seed quantity, and ground speed on the air-assisted high-speed precision seeding system. Dong et al. (2024) designed a new type of perforated precision seed metering device to improve the metering performance and energy efficiency of corn mechanical seed metering device under high-speed operation conditions, and the final performance coefficient was more significant than 9.2. Li et al. (2023a) designed a high-speed precision seed metering device for corn centrifugal grouting cleaning based on the principle of centrifugal force generated by the high-speed circular motion of seeds. Through response surface experiment optimization, it was found that the energy consumption during seeding is much lower than that of the air-suction seed metering device. Moreover, increasing the negative pressure improved the quality of feed index. Overall, it is evident that the working pressure of existing pneumatic maize metering devices exceeds 3 kPa, resulting in significant work energy consumption and a sharp increase in usage costs. Therefore, reducing the working pressure can effectively diminish production power consumption and contribute to the clean development of the maize industry.

To achieve efficient and clean corn planting operations, this study introduces a novel pneumatic maize metering device leveraging the principles of centrifugal force and variable-diameter structure. The device comprises three essential components: the seed supply device, the supply tube, and the maize metering device, forming an annular cavity via the hole insert, seeding plate, seed limit board, and front shelf. Utilizing high-speed rotational operations, seeds are propelled regularly through the airflow and hole insert within the annular cavity, simultaneously generating centrifugal force. As the seed limit board’s diameter abruptly changes, the typed hole insert collides with the seed limit board due to the centrifugal effect, disrupting the force chain among the seeds and altering their orientation in the hole insert to achieve optimal seed filling. To validate the performance and energy consumption of the seed metering device, a response surface test was conducted in a laboratory setting to evaluate the device’s operational efficiency. Subsequently, comprehensive energy consumption comparisons were made through experiments with different maize metering devices, verifying the energy consumption of the new maize metering device using the coefficient of performance (COP) as the evaluation metric.

## 2 Materials and methods

**Figure 1:**
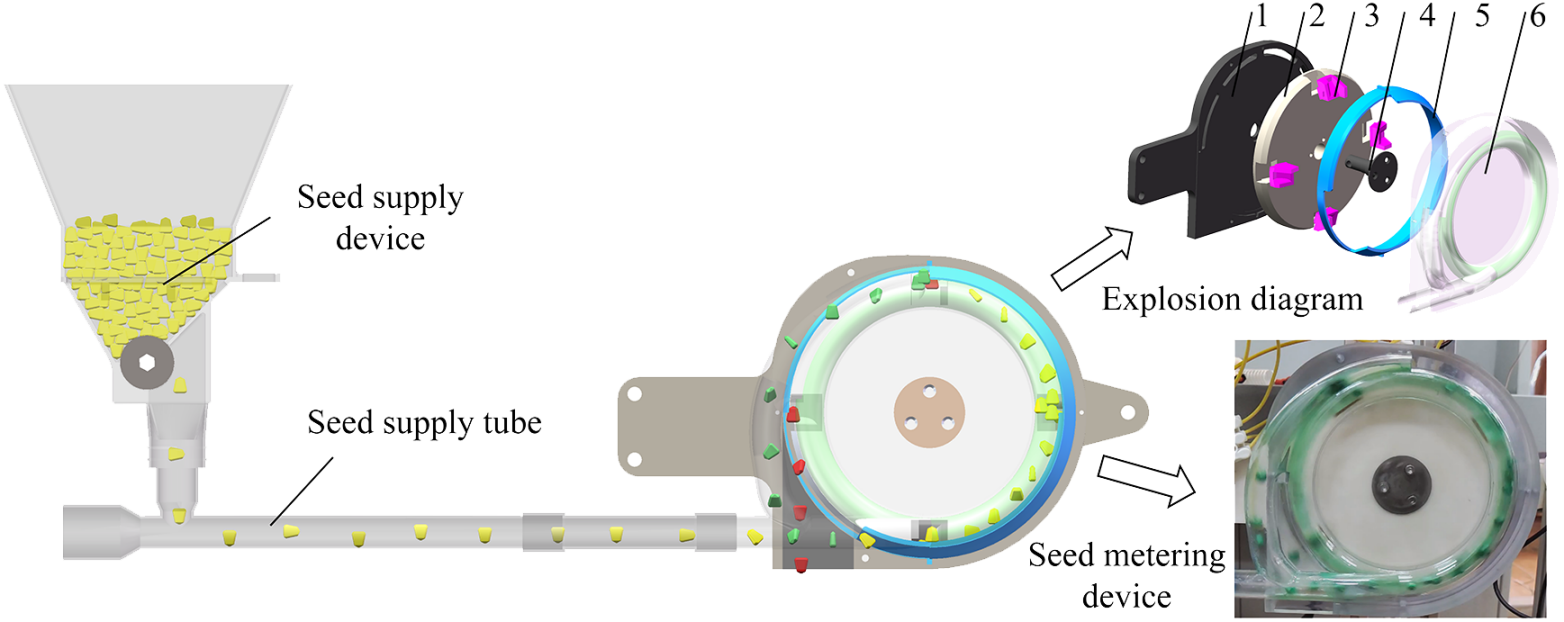
Structure of the seed metering system. 1. Rear shell; 2. Seeding plate; 3. Hole insert; 4. Shaft Bearing; 5. Seed limit board; 6. Integrated front shell.

The envisaged seed metering system is founded upon the governing principle of centrifugal force engendered by the circular trajectory of seeds. It predominantly comprises three integral components, as delineated in **Figure 1**. The seed metering device hinges upon the seed supply device and seed supply tube for the conveyance of seeds. The seeds descending from the seed supply device are systematically and uniformly propelled into the seed’s primary filling area by the airflow originating from the seed supply tube, thereby orchestrating a seamless seed flow.

### 2.1 Seed metering device

**Figure 2:**
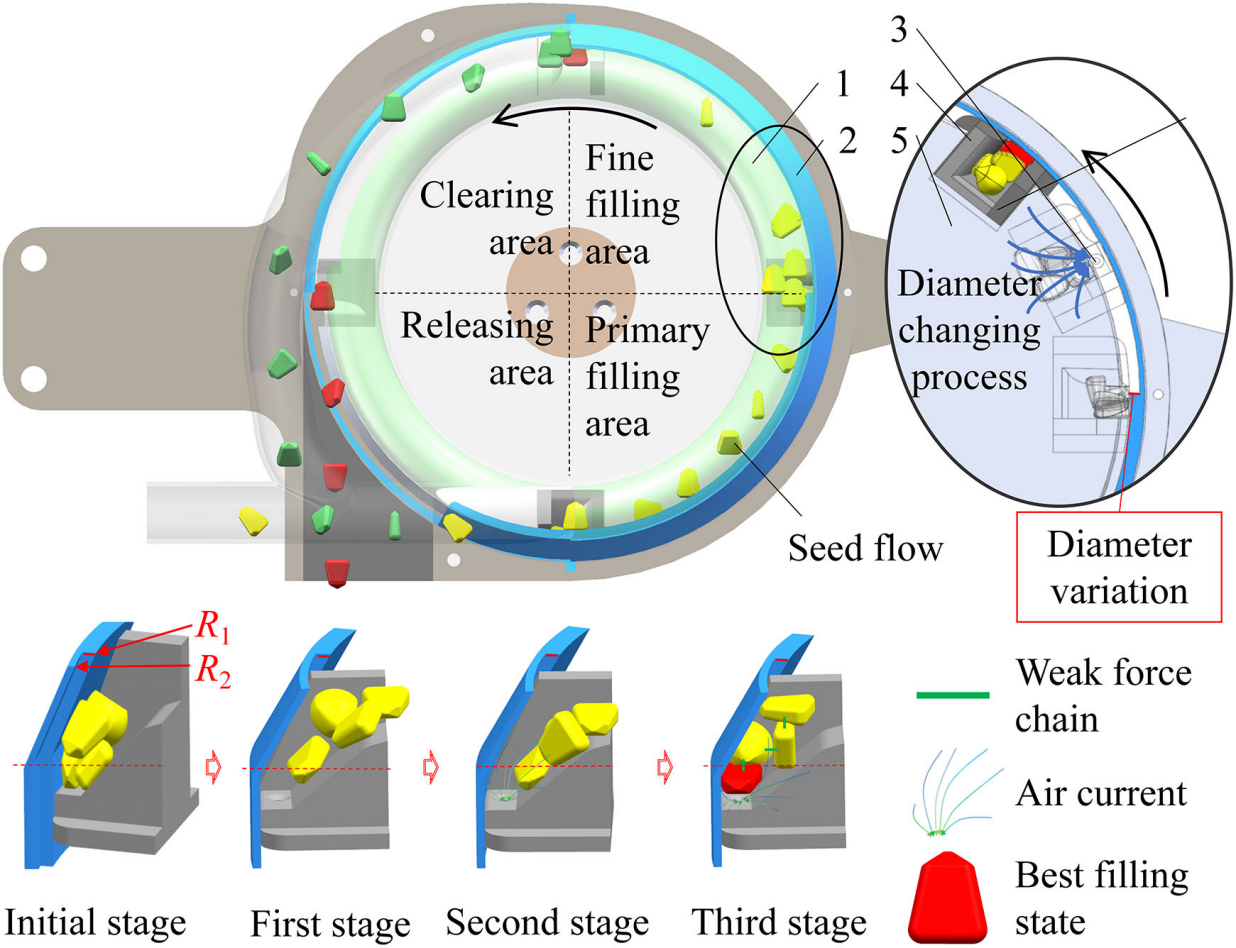
Working principle of the novel centrifugal variable-diameter type metering device. The green color represents excess filling seeds, the red color represents effective filling seeds, and the yellow color represents maize seeds. 1. Annular cavity; 2. Seed limit board; 3. Through-hole; 4. Hole insert; 5. Seeding plate.

According to the operational sequence of the seed metering device, the annular cavity undergoes a partition into the primary filling, fine filling, clearing, and releasing areas, as elucidated in **Figure 2**. In the primary filling area, the seeds accommodated within the hole insert rotate in the annular cavity. Subject to a more substantial centrifugal force, the groove for hole insert filling becomes susceptible to seed blockage or elevation. Therefore, in the fine filling area, bolstering the auxiliary airflow and modifying the thickness of the seed plate is imperative to ensure the quality of filling and accomplish stable grouting of individual seeds. This intricate process unfolds in three discernible stages. Initially, the interaction between the hole inserts and the seed limit board begets stimulating vibrations, disrupting the seed force chain within the hole and inducing seed cluster disarray. Subsequently, airflow is discharged through the through-hole. With the efflux of airflow, seeds proximate to the through-hole at the bottom are directed downward and firmly pressed by the airflow, affecting guidance and positioning. This ensures optimal seed placement for precise single-seed filling. In the final stage, the seeds atop the hole insert undergo pressure from the seeds filled at the bottom. This enhances seed-carrying stability, facilitating the attainment of single-seed precision filling. During the clearing area, the protective space of the seed limit board diminishes, causing unsupported seeds to be expelled from the hole insert due to centrifugal force. After the solitary seed-releasing area, the constraint surface on the seed limit board vanishes. The seed, disengaging from the hole insert through centrifugal force, achieves single-grain precision seeding. To avert irregular movements of cleared seeds, influencing seeding performance, these removed seeds are thrust into the external reflux tube by centrifugal force and airflow. They then return methodically along the tube to the primary filling area, contributing to the subsequent cycle of seed filling. Its minimal power consumption during operation significantly diminishes production and utilization costs, fostering a cleaner seeding process.

### 2.2 Design of critical components

#### 2.2.1 Front shell

**Figure 3:**
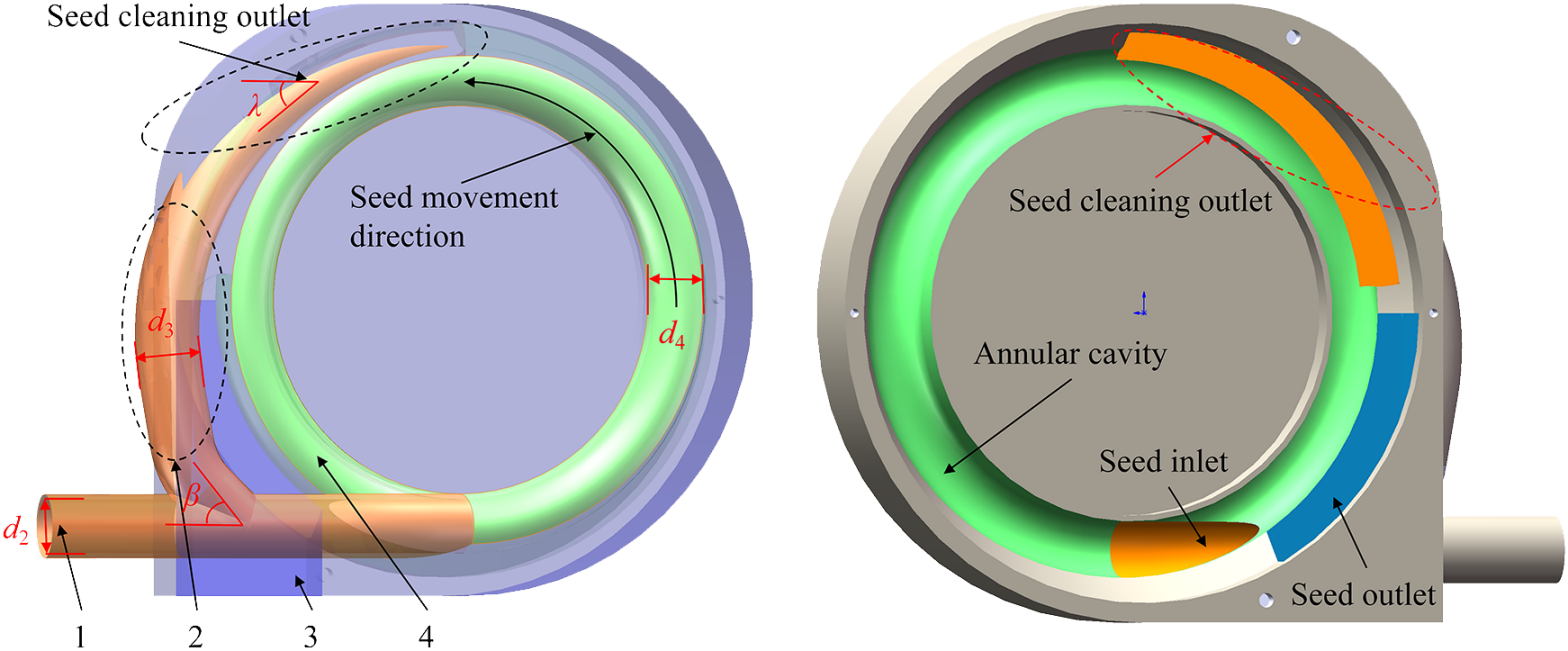
Sectional view of the front shell. 1. Seed inlet; 2. Reflux tube; 3. Seed outlet; 4. Annular cavity.

The seeds seamlessly orchestrate a stable flow within the annular cavity, culminating in meticulously executing the filling, clearing, and releasing processes. The front shell, depicted in **Figure 3**, encompasses key components such as a seed inlet, an annular cavity, a reflux tube, and a seed outlet. In a strategic move to curtail air consumption and seed rebound, the central region of the front shell is isolated. Simultaneously, an external reflux tube is ingeniously integrated to preempt the issues of reseeding seed rebound arising from the erratic movement of cleared seeds within the annular cavity. Ensuring a systematic reintroduction of cleared seeds into the primary filling area, this design also safeguards against the direct flow of airflow from the inlet tube to the reflux tube. The inner diameter is meticulously determined, ensuring it does not surpass the diameter of the inlet tube while forming an acute angle with it. In practical operations, the inner diameter of the annular cavity tube is calibrated to accommodate the hole insert, settled at 0.02 m. The inner diameters of the inlet tube and the reflux tube are harmonized with the annular cavity tube to avert airflow reflux, all fixed at 0.02 m, with a reflux angle *β* set at 45°.

The cleaning duration is compressed during high-speed operations, often resulting in the ineffective removal of surplus seeds. Hence, it becomes imperative to design the cleaning outlet’s position meticulously. The board’s width is deliberately reduced in the clearing area, causing excess seeds to lose support from the board. Subject to the influence of centrifugal force, the seed undergoes a primary detachment from the hole insert, followed by a streamlined throwing motion. During high-speed operation, the centrifugal force exerted on the seed significantly surpasses that of gravity, warranting the dismissal of gravity’s influence. The seeding plate’s rotation angle (*δ*) upon seed separation from the hole insert can be calculated using its motion equation, elegantly illustrated in Equation 1.


(1) $$\delta =\sqrt{\frac{2L_{1}}{r}}$$

It can be seen from Equation 1 that the rotation angle (*δ*) when the seed is separated from the mold hole is only determined by the variable (*L*_1_), the distance, and the radius (*r*) of the seed hole insert. Remarkably, this angle remains independent of the rotation speed. Consequently, alterations in ground speed will not induce changes in the cleaning point’s position. Following meticulous calculations, the angle (*δ*) is estimated to be approximately 30°. The cleaning port of the reflux tube is strategically positioned at the tangent of the cleaning point, forming an angle (*λ*) of 60°.

#### 2.2.2 Hole insert

**Figure 4:**
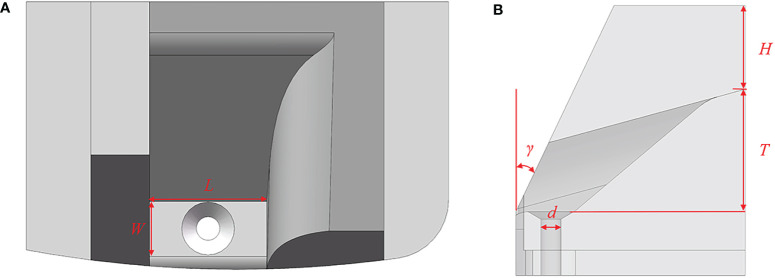
Hole insert schematic diagram. **(A)** Top view; **(B)** Perspective view.

The hole insert comes into direct contact with the seed, rendering its structural form and parameters pivotal in shaping its performance. As shown in **Figure 4**.

**Figure 5:**
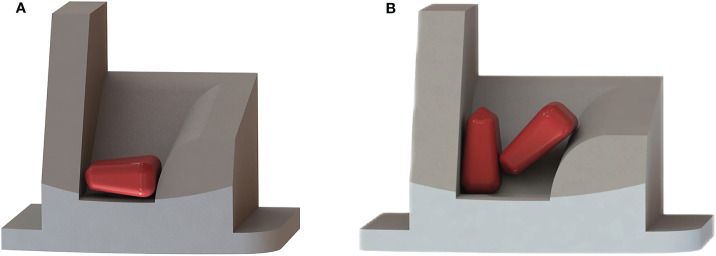
The different states of the seed in the hole insert. **(A)** Ideal filling posture; **(B)** Schematic of seeds jamming.


The contact area between the reclined position of seeds and the specific hole is maximized, offering a relatively stable posture conducive to efficient filling. Hence, a higher proportion of reclined postures during filling corresponds to superior seeding performance, as depicted in **Figure 5A**. The hole must accommodate three to five seeds to ensure the primary filling effect. Influenced by centrifugal force, the seeds above the hole exert pressure, covering the seeds within the effective filling area at the hole’s base. However, an excess of seeds poses challenges for clearing. Therefore, the critical dimensions of the hole insert should satisfy the following Equation 2:

(2) $$\left\{ \begin{array}{l} l_{0}<L<l_{0}+t_{0}\\ w_{0}<W<w_{0}+t_{0}\\ l_{0}<T<1.5l_{0}\\ H>0.5l_{0} \end{array}\right.$$

After careful deliberation, the hole *L*, *W*, *T*, and *H* dimensions are chosen as 0.012, 0.009, 0.012, and 0.008 m, respectively. In collaboration with the seed limit board to facilitate the seamless sliding of seeds into the hole, the *γ* is set at 30°.

Owing to the diverse shapes and substantial differences in size across the three axes, they intertwine during the filling process, leading to frequent irregular clustering of multiple seeds at the hole’s base—a situation prone to jamming, as shown in **Figure 5B**. Consequently, following the completion of the primary filling process, the seed orientation within the effective filling area undergoes adjustment to a singular reclined posture through variable-diameter and airflow-guided positioning. The seeds at the hole were positioned through the pressure generated by turbulence resistance (Kabeel et al., 2019). So, a through-hole is incorporated at the bottom of the hole insert to achieve airflow guidance and precise positioning. Research has determined that the chamfered through-hole exhibited a wide range of airflow, which facilitates the adjustment of seed orientation (Li et al., 2021). Consequently, the chamfered through-hole has been chosen for this study. Proximate to the through-hole, seeds gradually encounter airflow, enabling dynamic adjustment of their orientation and position during this process. Following the design manual of agricultural machinery, select a through-hole with a diameter (*d*) of 0.004 m (Ji et al., 2018).

#### 2.2.3 Seeding plate

**Figure 6:**
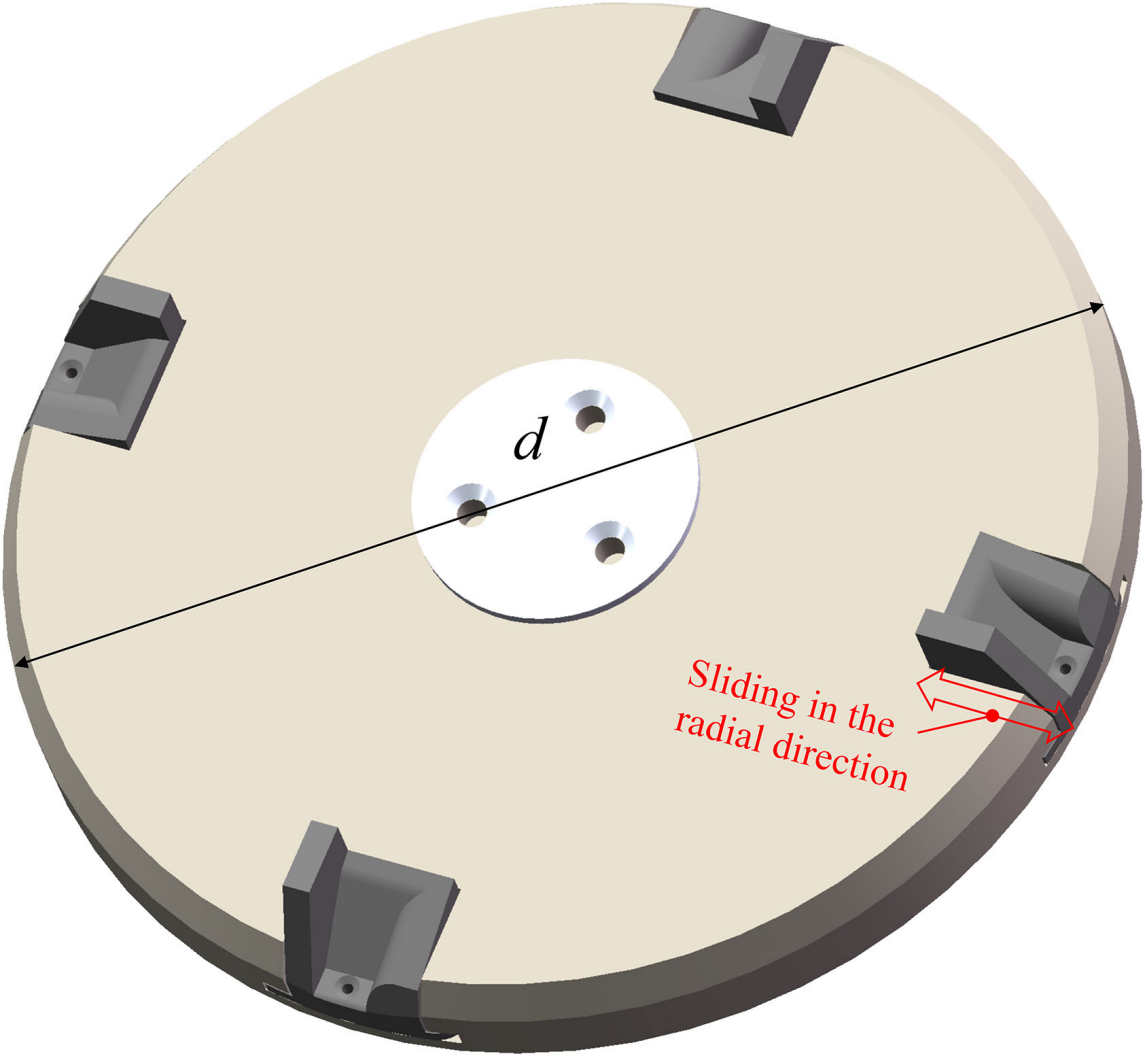
Schematic diagram of the seeding plate structure.

The seeding plate serves as a rotating component, propelling seeds in a rapid circular motion. A sliding groove is incorporated into the seeding plate to achieve the centrifugal variable diameter feature of the hole insert, as illustrated in **Figure 6**.

The duration of the filling process directly impacts the success rate of sowing. The plate’s diameter significantly influences the performance and dimensions upon determining the hole number. Nevertheless, an excessive diameter for the seeding plate may result in increased dimensions, manufacturing costs, and assembly challenges for the seeding device. Referring to the investigations conducted by Gao et al. (2023), which featured a seeding plate with four-hole inserts, the diameter is conclusively established as 0.20 m.

#### 2.2.4 Seed limit board

The seed limit board is crucial in achieving variable-diameter single-grain fine filling, ensuring precision in seeding, and seamlessly integrating with the hole insert. Specifically, the radius *R*_2_ (large arc) of the seed limit board in the primary filling area is larger than the radius *R*_1_ (small arc) in the fine filling area. A radial mutation occurs at the junction between the seed limit board in the primary filling area and the seed limit board in the fine filling area. Its primary function is to expel the insert from the hole, allowing seeds experiencing excessive pressure during the primary filling process to loosen.

**Figure 7:**
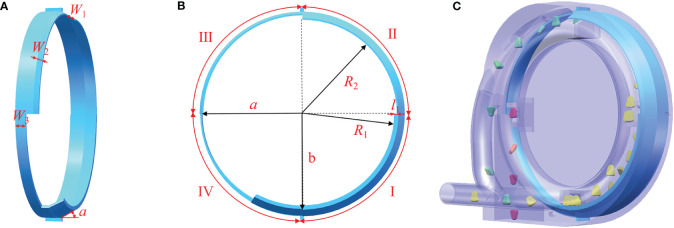
Structure diagram of the seed limit board. I. Primary filling area; II. Fine filling area; III. Clearing area; and IV. Releasing areas. **(A)** Side view; **(B)** Front view; **(C)** Simulating.

The seed limit board incorporates a gradient curve structure, facilitating the gradual repositioning of the hole insert to its original state. Guided by the contour curve, the structural configuration of the seed limit board is meticulously determined, involving essential parameters such as the front width *W*_1_ in the primary filling and fine filling areas, the front width *W*_2_ in the cleaning area, the width *W*_3_ in the releasing area, the front angle *α*, the radial mutation length *l*_1_, the small arc radius *R*_1_, and the large arc radius *R*_2_. A collaborative synergy unfolds between the seed limit board and the hole insert in the intricate seed-filling process. After thorough consideration, the *W*_1_, *W*_2_, *W*_3_, *α*, and *l*_1_ are determined as 0.017 m, 0.006 m, 0.010 m, 30°, and 0.003 m, respectively. The *R*_1_ is set at 0.101 m to harmonize with the seed limit board, while the maximum *R*_2_ reaches 0.104 m, as illustrated in **Figure 7**.

### 2.3 Experiments and methods

#### 2.3.1 Experimental bench

**Figure 8:**
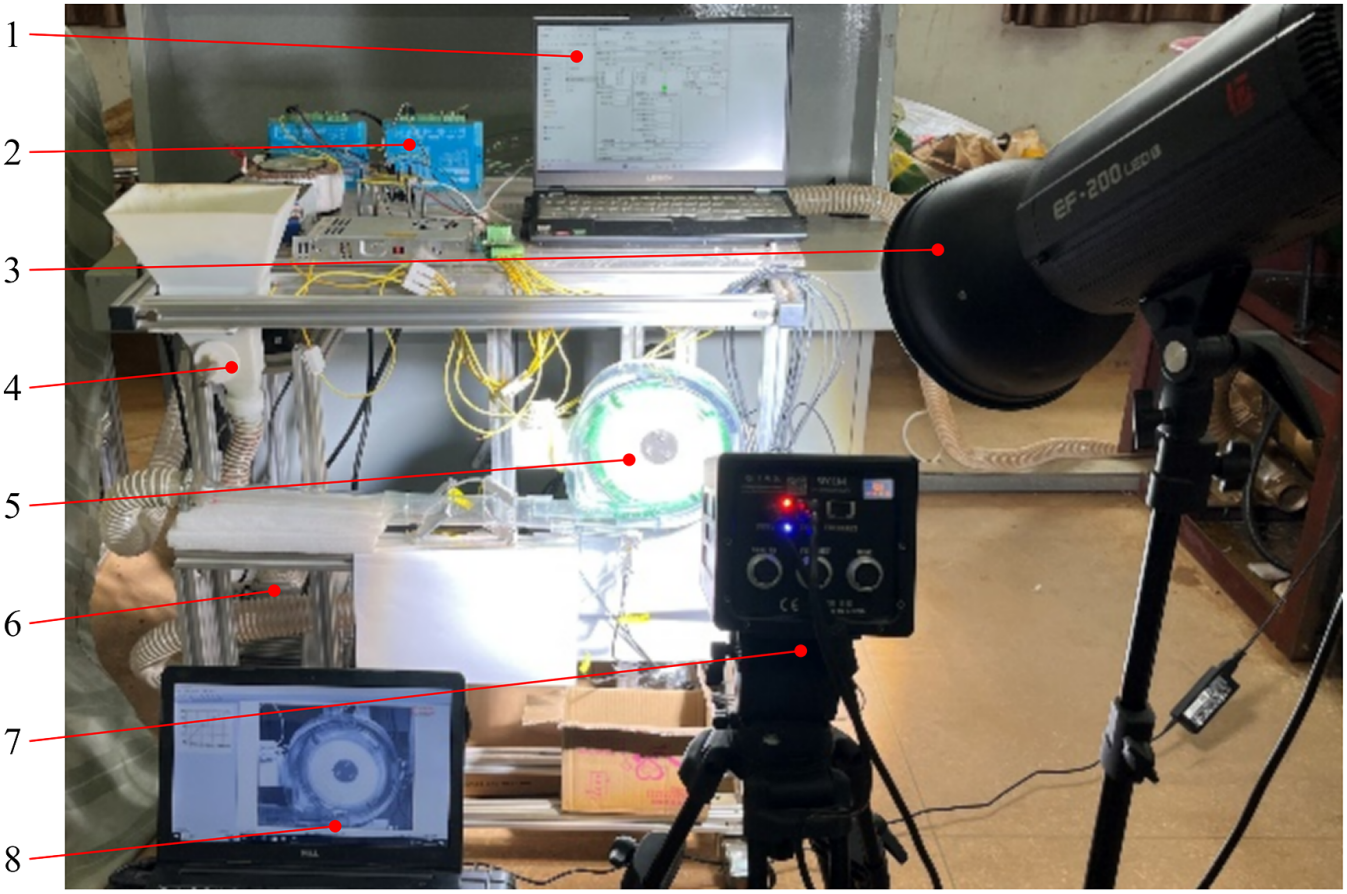
The experimental bench. 1. PC to control the seed metering device; 2. Motor controller; 3. Fill light; 4. Seed feeding device; 5. Seed metering device; 6. Turbine blower; 7. High-speed camera; 8. PC to control the high-speed camera.

We meticulously engineer an experimental bench to evaluate the consumption of seeding performance and substantiate the efficacy and superiority of the proposed maize metering device. This experimental bench includes a seed metering system, a high-speed camera (HF Agile Device Co., Ltd., China), two motors, and two motor controllers, showcased in **Figure 8**. The seeding system underwent trial production utilizing 3D printing technology and cost-effective resin materials. Zhengdan-958 maize (ungraded) seeds are utilized in the experiment, maintaining a plant spacing of 0.30 m during seeding (Gao et al., 2020).

#### 2.3.2 Response surface experiment of CCD

The quality of the metering device lies in its high-speed adaptability across varying ground speeds. Furthermore, the pneumatic metering device’s wind pressure significantly influences the velocity of seeds within the apparatus, and maintaining an appropriate seed-feeding rate is crucial for sustaining the dynamic equilibrium. The optimal interplay among the factors mentioned is pivotal for achieving high-speed precision seeding devices. Consequently, the experimental factors encompass ground speed (*X*_1_), wind pressure (*X*_2_), and feeding rate (*X*_3_). Adhering to the international standard ISO 7256/1, each experiment involves sowing 500 seeds. Equation 3 below presents the formula for computing these experimental indicators.


(3) $$\left\{ \begin{array}{l} Y_{1}=\frac{n_{1}}{N'}\times 100\%\\ Y_{2}=\frac{n_{2}}{N'}\times 100\%\\ Y_{3}=\frac{n_{0}}{N'}\times 100\% \end{array}\right.$$

where *Y*_1_ denotes the quality of feed index, %; *Y*_2_ denotes the multi-index, %; *Y*_3_ denotes the miss index, %; *n*_1_ denotes the number of seeds normally sown; *n*_2_ denotes the number of multiples; *n*_0_ denotes the number of misses; *N*’ denotes the number of intervals.

Response surface experiments are pivotal in assessing factors’ influence on performance (Gao et al., 2021; Liu et al., 2023; Kadam and Pawade, 2024). The experiments encompassed three factors at five levels. The experimental design process and the subsequent result processing are carried out using Design-Expert 13 (Stat-Ease, Minneapolis, Minnesota, USA). A regression analysis is also performed to ascertain the optimal operational parameters, as shown in **Table 1**.

**Table 1:** Levels of the experimental factors.

Level	Factors		
	Ground speed *X*_1_ (km/h)	Wind pressure *X*_2_ (kPa)	Feeding rate *X*_3_ (seeds/s)
-1.682	12.0	1.0	10
-1	12.8	1.0	14
0	14.0	1.5	20
1	15.2	1.8	26
1.682	16.0	2.0	30

#### 2.3.3 Performance and energy efficiency experiments

**Figure 9:**
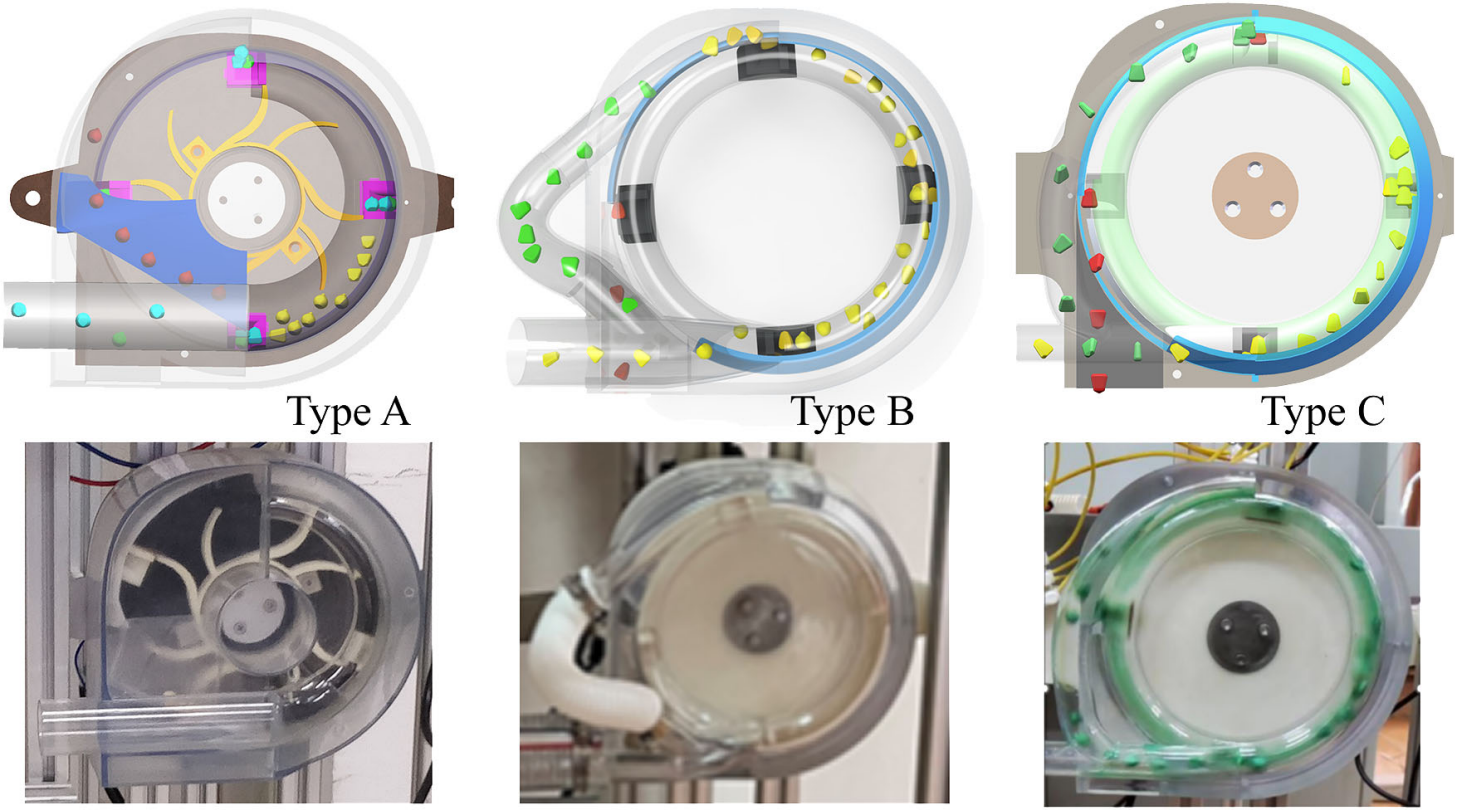
The experimental seed metering devices. The *d*_a_ of Type A is 0.043 m; the *d*_a_ of Type B is 0.036 m; the *d*_a_ of Type C is 0.020 m.


Maintaining optimal wind pressure is crucial for ensuring the efficient and regular operation of the pneumatic planter. Reduced wind pressure lowers production energy consumption and enhances energy efficiency, enabling low-energy and clean production. A comparative analysis is conducted with similar centrifugal seed metering devices to elucidate the power consumption of the novel centrifugal variable-diameter pneumatic seeding device, as depicted in **Figure 9**. Notably, Type A represents a high-speed centrifugal filling-cleaning precision seed metering device. It utilizes a diversion turbine to disrupt the seed population, ensuring optimal filling, and employs an internal reflux replication scheme for seed reflow refilling (Zhao et al., 2024). Type B represents the previous generation of the centrifugal variable diameter pneumatic seed metering device. It relies on a distinctive variable diameter structure for optimal filling and utilizes an external reflux tube for reflow refilling the cleaned seeds (Gao et al., 2022a). Type C represents the novel centrifugal variable-diameter type metering device designed. It reduces the inner diameter of the annular tube and increases the turning radius of the reflux tube. The performance, power consumption, and COP of three distinct types of centrifugal filling-cleaning seed metering devices are assessed under optimal operating conditions, corresponding to ground speeds of 12, 14, 16, and 18 km/h. The power needed to generate wind pressure can be computed using Equation 4, and the COP can be determined using Equation 5.

(4) $$P=\frac{1}{4}\pi d_{a}^{2}v_{a}p$$

(5) $$\text{COP}=\frac{A}{P}$$

where *P* is the power required to generate wind pressure (kW), *d*_a_ is the diameter of the airflow tube opening (m), *v*_a_ is the air velocity (m/s), and *p* is the wind pressure (kPa).

## 3 Results and discussion

### 3.1 Analysis of variance

**Table 2** displays the results of the laboratory bench experiment. Subsequently, distinct mathematical models are formulated linking ground speed, wind pressure, and feeding rate with the different indexes. Linear regression equations for *Y*_1_, *Y*_2_, and *Y*_3_ are derived individually. Following the elimination of non-significant factors, the regression equation for the quality of feed index is formulated, as illustrated in Equation 6.

**Table 2:** Experimental scheme and results.

No.	Factors			Performance indexes		
	*X*_1_ (km/h)	*X*_2_ (kPa)	*X*_3_ (seeds/s)	*Y*_1_ (%)	*Y*_2_ (%)	*Y*_3_ (%)
1	12.8	1.2	14	90.93	6.27	2.80
2	15.2	1.2	14	88.53	8.54	2.93
3	12.8	1.8	14	89.33	8.14	2.53
4	15.2	1.8	14	88.53	8.80	2.67
5	12.8	1.2	26	94.13	5.07	0.80
6	15.2	1.2	26	90.67	8.00	1.33
7	12.8	1.8	26	90.13	8.67	1.20
8	15.2	1.8	26	89.60	6.93	3.47
9	12.0	1.5	20	91.33	7.33	1.33
10	16.0	1.5	20	87.87	8.93	3.20
11	14.0	1.0	20	92.80	6.53	0.67
12	14.0	2.0	20	89.07	8.27	2.67
13	14.0	1.5	10	88.67	7.60	3.73
14	14.0	1.5	30	92.60	5.13	2.27
15	14.0	1.5	20	93.87	5.33	0.80
16	14.0	1.5	20	92.27	6.13	1.60
17	14.0	1.5	20	93.20	5.07	1.73
18	14.0	1.5	20	92.40	5.93	1.67
19	14.0	1.5	20	94.00	4.87	1.13
20	14.0	1.5	20	94.93	4.13	0.93
21	14.0	1.5	20	93.20	5.07	1.73
22	14.0	1.5	20	93.07	5.53	1.40
23	14.0	1.5	20	92.53	5.73	1.93


(6) $$\begin{array}{l} Y_{1}=93.27-0.95X_{1}-0.95X_{2}+1.01X_{3}+0.57X_{1}X_{2}-1.30X_{1}{\;}^{2}-0.82X_{2}{\;}^{2}-0.93X_{3}{\;}^{2}\\ Y_{2}=5.31+0.50X_{1}+0.56X_{2}-0.53X_{3}-0.79X_{1}X_{2}+1.02X_{1}{\;}^{2}+0.77X_{2}{\;}^{2}+0.40X_{3}{\;}^{2}\\ Y_{3}=1.45+0.46X_{1}+0.39X_{2}-0.48X_{3}+0.32X_{1}X_{3}+0.38X_{2}X_{3}+0.27X_{1}{\;}^{2}+0.53X_{3}{\;}^{2} \end{array}$$

The variance analysis quality of the feed index experiment data is conducted using the software. The significance of the experiment results for the regression equation is presented in **Table 3**. A *P*-value less than 0.05 for the model suggests its significance. A lack-of-fit *P*-value exceeding 0.1 implies insignificance in the lack of fit. The impact of each factor on the quality of feed index, ranked from largest to smallest, is *X*_3_, *X*_1_, and *X*_2_. The influence of each factor on the multi-index, from largest to smallest, is *X*_2_, *X*_3_, and *X*_1_. Among these, the first-order terms (*X*_1_, *X*_2_, *X*_3_), the interaction term (*X*_1_ *X*_2_), and the quadratic term (*X*_1_^2^, *X*_2_^2^, *X*_3_^2^) significantly affect *Y*_1_ and *Y*_2_, while the others are not significant. The influence of various factors on the miss index is ranked from largest to smallest: *X*_3_, *X*_1_, and *X*_2_. The first-order terms (*X*_1_, *X*_2_, *X*_3_), the interaction terms (*X*_1_ *X*_3_, *X*_2_ *X*_3_), and the quadratic terms (*X*_1_^2^, *X*_3_^2^) significantly affect the miss index, while the others are not significant.

**Table 3:** Results of the analysis of variance for quadratic models.

Source	*Y*_1_		*Y*_2_		*Y*_3_	
	*F*-value	*P*-value	*F*-value	*P*-value	*F*-value	*P*-value
Mode	19.92	<0.0001**	14.03	<0.0001**	11.36	<0.0001**
*X*_1_	23.80	0.0003**	9.53	0.0087**	17.94	0.0010**
*X*_2_	23.56	0.0003**	11.82	0.0044**	13.41	0.0029**
*X*_3_	26.86	0.0002**	10.75	0.0060**	20.15	0.0006**
*X*_1_X_2_	4.93	0.0448*	13.83	0.0026**	2.43	0.1431
*X*_1_X_3_	0.15	0.7049	1.06	0.3216	5.08	0.0422*
*X*_2_X_3_	2.89	0.1129	0.056	0.8165	7.47	0.0171*
*X*_1_^2^	51.24	<0.0001**	46.80	<0.0001**	7.35	0.0178*
*X*_2_^2^	20.71	0.0005**	26.20	0.0002**	0.36	0.5595
*X*_3_^2^	26.39	0.0002**	7.16	0.0191*	28.31	0.0001**
Lack of fit	0.204	0.9517	0.8855	0.5323	1.40	0.3188

* represents *P*< 0.05, indicating that the model term is significant, while ** represents *P*< 0.01, indicating that the model term is extremely significant.

### 3.2 Response analysis of the experimental factors

**Figure 10:**
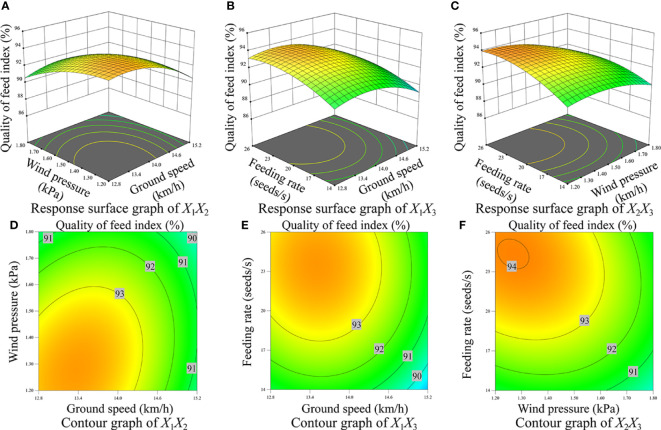
Response surface diagram and contour diagram of interaction effect on the quality of feed index. **(A)** Response surface diagram of interaction between wind pressure and ground speed, **(B)** Response surface diagram of interaction between feeding rate and ground speed, **(C) ** Response surface diagram of interaction between feeding rate and wind pressure, **(D)** Contour diagram of interaction between wind pressure and ground speed, **(E)** Contour diagram of interaction between feeding rate and ground speed, **(F)** Contour diagram of interaction between feeding rate and wind pressure.

**Figure 11:**
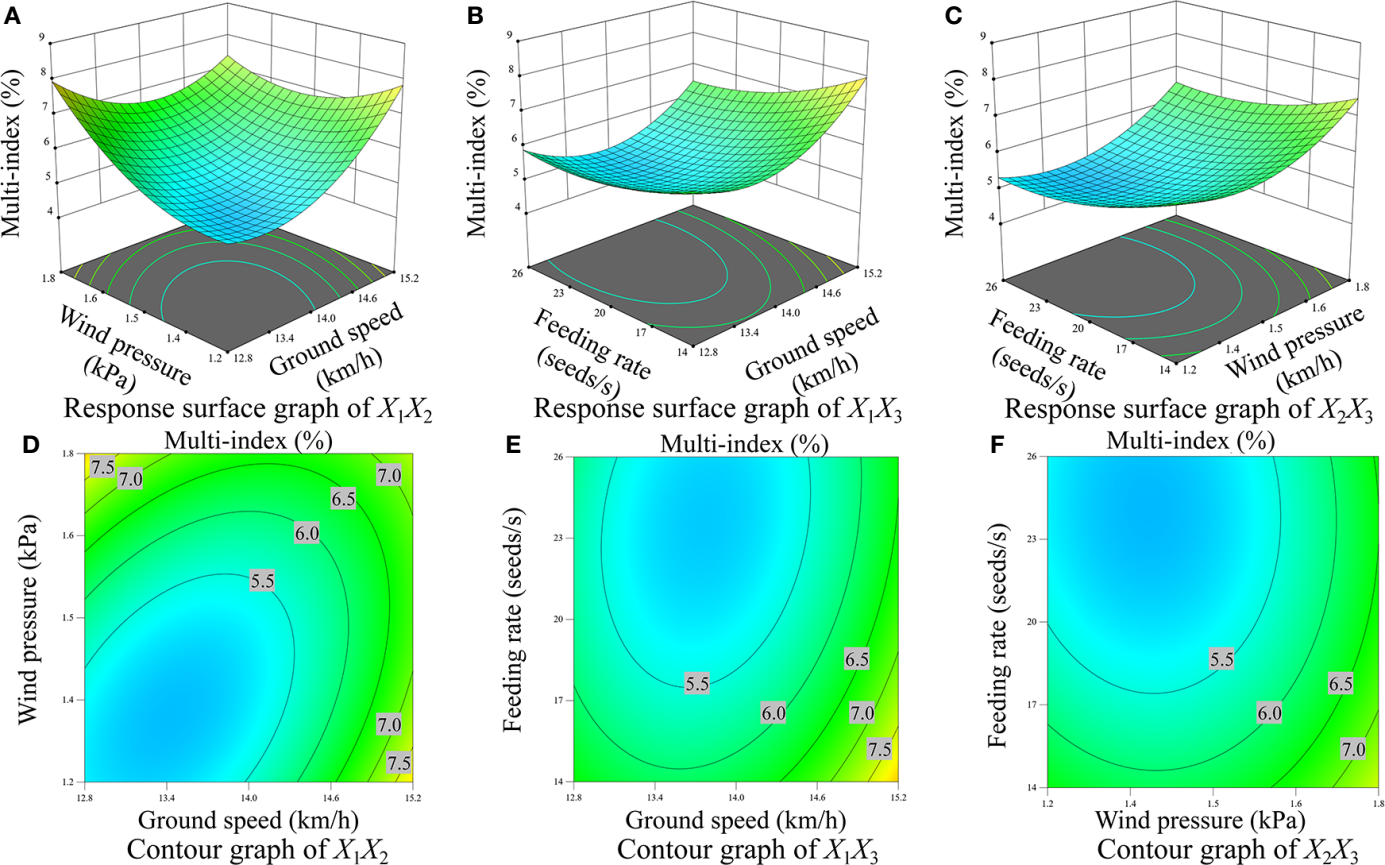
Response surface diagram and contour diagram of interaction effect on the multi-index. **(A)** Response surface diagram of interaction between wind pressure and ground speed, **(B)** Response surface diagram of interaction between feeding rate and ground speed, **(C)** Response surface diagram of interaction between feeding rate and wind pressure, **(D)** Contour diagram of interaction between wind pressure and ground speed, **(E)** Contour diagram of interaction between feeding rate and ground speed, **(F)** Contour diagram of interaction between feeding rate and wind pressure.

**Figure 12:**
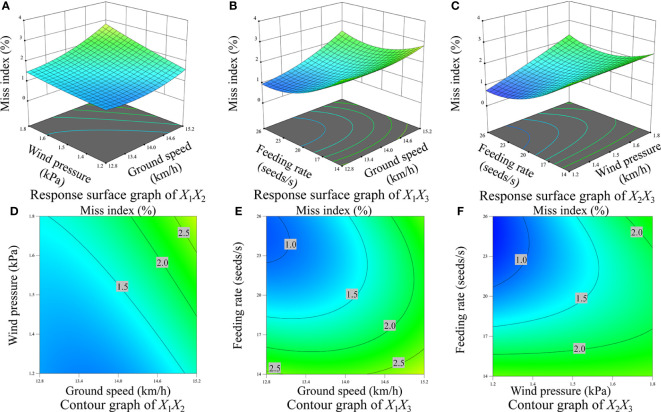
Response surface diagram and contour diagram of interaction effect on the miss index. **(A)** Response surface diagram of interaction between wind pressure and ground speed, **(B)** Response surface diagram of interaction between feeding rate and ground speed, **(C)** Response surface diagram of interaction between feeding rate and wind pressure, **(D)** Contour diagram of interaction between wind pressure and ground speed, **(E)** Contour diagram of interaction between feeding rate and ground speed, **(F)** Contour diagram of interaction between feeding rate and wind pressure.

**Figures 10**, **11**, **12A, D** display the surface and contour drawings illustrating the impact of the interaction term *X*_1_ *X*_2_ on the different indexes at *X*_3_ of 20 seeds/s. The centrifugal force of the seed increases as the *X*_1_ increases, leading to an enhanced filling effect. Nevertheless, when the *X*_1_ exceeds 14.0 km/h, excessive centrifugal pressure is applied to the seeds, compressing more than one seed at the bottom and impeding effective removal during the cleaning process. This leads to a reduction in *Y*_1_. Conversely, when the *X*_2_ exceeds 1.5 kPa, the inlet velocity of seeds rises due to high wind pressure, causing some seeds to be unable to fill into the hole insert and be discharged directly. This leads to a reduction in *Y*_1_. When *X*_3_ is 20 seeds/s, maintaining the *X*_2_ between 1.2~1.5 kPa and the *X*_1_ between 12.8~14.0 km/h, the *Y*_1_ can be upheld above 93.00%.

**Figures 10**, **11**, **12B, E** depict the surface and contour drawings illustrating the influence of the interaction term *X*_1_ *X*_3_ on the different indexes when the *X*_2_ is 1.5 kPa. In cases where the *X*_1_ and *X*_3_ are both low, fewer seeds are in the hole insert, leading to a lower *Y*_1_. Conversely, with high *X*_1_ and low *X*_3_, excessive centrifugal force intensifies the impact vibration effect on the hole at the variable diameter position of the seed limit board. Consequently, the seeds initially filled in the hole insert become prone to detachment, leading to a lower *Y*_1_. When the *X*_1_ and *X*_3_ are high, numerous seeds get compressed at the bottom. The cleaning process, however, fails to effectively eliminate the excess seeds, leading to a decline in *Y*_1_. When *X*_2_ is 1.5 kPa, maintaining the *X*_3_ between 20~26 seeds/s and the *X*_1_ between 12.8~14.0 km/h, the *Y*_1_ can be sustained at levels above 93.00%.

**Figures 10**, **11**, **12 C, F** depict the surface and contour drawings illustrating the influence of the interaction term *X*_2_ *X*_3_ on the different indexes when the *X*_1_ is 14.0 km/h. When *X*_2_ and *X*_3_ are both low, fewer seeds are filled into the hole insert, and the airflow is weak, leading to lower *Y*_1_ and *Y*_2_, and *Y*_3_ is the opposite. When the *X*_3_ is low and the *X*_2_ is high, it leads to a poor filling effect and, consequently, a lower *Y*_1_. When the *X*_3_ and *X*_2_ are high, the occurrence of seeds falling directly from unfilled holes increases, resulting in a decrease in the *Y*_1_. With *X*_1_ at 14 km/h, maintaining the *X*_2_ between 1.20 and 1.50 kPa, and *X*_3_ between 20 and 26 seeds/s, *Y*_1_ can surpass 93.00%.

### 3.3 Parameter optimization

The response surface graph indicates an optimum value for the seeding. Utilizing regression equations, the optimal combination of *X*_3_, *X*_2_, and *X*_1_ for seeding performance can be determined by optimizing the *Y*_1_, *Y*_2_, and *Y*_3_. The optimization objectives include achieving the lowest *Y*_2_ and *Y*_3_ and the highest *Y*_1_. Conducting a multi-factor optimization solution on the established quadratic regression model is performed under specified boundary conditions, with constraint conditions outlined as illustrated in **Equation 7**.


(7) $$\left\{ \begin{array}{l} \max Y_{1}\left(x_{1},x_{2},x_{3}\right)\\ \min Y_{2}\left(x_{1},x_{2},x_{3}\right)\\ \min Y_{3}\left(x_{1},x_{2},x_{3}\right)\\ 12\text{ km/h}\leq x_{1}\leq 16\text{ km/h}\\ 1\text{ kPa}\leq x_{2}\leq 2\text{ kPa}\\ 10\text{ seeds/s}\leq x_{3}\leq 30\text{ seeds/s} \end{array}\right.$$

Design-Expert calculations deduce that the highest seeding performance occurred at *X*_1_ of 13.2 km/h, *X*_2_ of 1.2 kPa, and *X*_3_ of 25 seeds/s. Under these conditions, the *Y*_1_, *Y*_2_, and *Y*_3_ are 94.45%, 5.02%, and 0.53%, respectively. Bench experiments are conducted under identical conditions to validate the accuracy of the optimal solution. The bench experiment results demonstrated that *Y*_1_, *Y*_2_, and *Y*_3_ are 95.20%, 3.87%, and 0.93%, respectively. These values closely align with the optimal solution, affirming their high reliability.

### 3.4 Performance and energy efficiency comparison results

#### 3.4.1 Operation performance

The seeding performance of three distinct centrifugal seed metering devices at varying ground speeds is detailed in **Table 4**. Evidently, the novel centrifugal variable-diameter type metering device effectively harnesses the centrifugal force generated during high-speed operation, exhibiting superior performance under optimal working conditions and ensuring *Y*_1_ exceeds 93.00%. Among these, the Type A seed metering device attains a higher wind pressure and *Y*_2_ under identical conditions. In the operation process, the metering device’s internal cavity relies on the wind pressure and the guide turbine structure to adjust the seed posture, and there is no airflow tube inside, so a large wind pressure is needed to ensure the wind pressure inside the whole metering device. At the same time, under the combined action of a larger wind pressure and a high-speed rotating diversion turbine structure, it is easy to make the seeds filled in the cavity directly discharged from the seed outlet so that the *Y*_2_ increases. Furthermore, seeds cleaned in the clearing area collide with the inclined baffle during the falling process, inducing irregular seed movement and increasing the *Y*_2_. The Type B seed metering device exhibits a relatively low *Y*_1_ and significant *Y*_2_ and *Y*_3_. The diameter of the annular seed tube in the seed metering device is large, which leads to the seeds not being filled with the hole insert directly ejected from the releasing area, and the *Y*_2_ is improved. Additionally, seeds cleaned in the clearing area, influenced by centrifugal force during the reflux tube, experience substantial inertial motion. The inadequate turning radius of the reflux tube is prone to blockages during reflux, diminishing the seed supply and subsequently elevating the *Y*_3_. The Type C seed metering device is optimized based on Types A and B. Compared to the Type A device, its *Y*_3_ increases, attributed to the collision strength between the hole insert and the seed limit board during the diameter-changing process. While this collision may lead to occasional miss-seeding, it aligns with the seed metering device’s design requirements. Regarding *Y*_1_ and *Y*_2_, Type C outperforms Type B across all metrics, requiring the least wind pressure and better aligning with cleaner production operational requirements.

**Table 4:** The experiment results of different types of seed metering devices.

Type	*X*_1_ (km/h)	*X*_2_ (kPa)	Speed of the plate (rpm)	*Y*_1_ (%)	*Y*_2_ (%)	*Y*_3_ (%)
Type A	12	9.2	166.67	90.35	8.91	0.74
	14	10.0	194.44	90.17	9.30	0.53
	16	11.3	222.22	90.47	8.86	0.67
	18	12.0	250.00	90.6	8.93	0.47
Type B	12	3.3	166.67	86.8	6.13	7.07
	14	3.5	194.44	86.13	7.74	6.13
	16	3.7	222.22	87.33	7.27	5.40
	18	3.9	250.00	85.52	8.72	5.76
Type C	12	1.3	166.67	93.87	5.46	0.67
	14	1.5	194.44	94.15	4.67	1.18
	16	1.7	222.22	93.36	5.38	1.26
	18	1.9	250.00	93.21	5.26	1.53

#### 3.4.2 Operation energy efficiency

**Figure 13:**
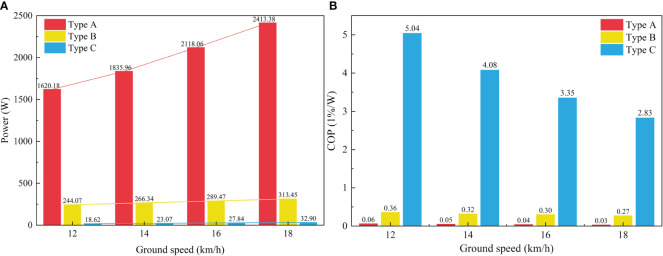
Comparison results of energy efficiency. **(A)** Power of seed metering devices; **(B)** COP of seed metering devices.

In **Figure 13A**, the power of each type at different ground speeds is illustrated, with the power ranking as Type A, Type B, and Type C from large to small. The power of all seed metering devices increases with the increase in ground speed, but the power consumption of Type A devices increases more obviously. In addition, the power consumption of Type A is significantly higher than that of Types B and C. The air annular tube design in the metering device cavity significantly reduces the required air pressure. The actual power consumption of Types B and C is about 85.00% and 98.00% lower than that of Type A, respectively, indicating that a suitable air annular tube helps similar metering devices reduce power consumption. The COP of each type of seed metering device at different ground speeds is presented in **Figure 13B**, where Type C, Type B, and Type A follow the sequence from large to small. The COP of Type C is about 86 times and 12 times that of Types A and B, respectively. The changing trend contrasts with power, with COP values decreasing as ground speed increases. The Type C seed metering device attains a higher COP value, signifying that the seed metering device designed excels in energy efficiency and is well-suited for maize’s efficient and clean production needs.

## 4 Conclusion

This study introduces a novel centrifugal variable-diameter metering device. The proposed seed metering device features a simpler structure and lower production costs. It requires only a small airflow during the operation process, leading to low energy consumption and a higher energy utilization rate, aligning more closely with the development trend of efficient and clean maize production.

(1) A novel centrifugal variable-diameter type metering device has been meticulously designed. The centrifugal diameter-changing effect and airflow-guided positioning facilitate the achievement of single-grain precision filling. The cleared seeds are systematically refilled through the outer filling of the shell, enabling high-speed precision seeding operations. The structure is streamlined, resulting in a simpler design and a significantly lower production cost.

(2) The regression orthogonal rotation combination experiment uses the self-built experiment platform. The response surface method is employed to elucidate the impact of each factor on the experiment index. The optimal seeding performance is achieved when the ground speed is 13.2 km/h, the wind pressure is 1.2 kPa, and the refilling amount is 25 seeds/s. During this optimal condition, the quality of feed index reaches 95.20%, the multi-index is 3.87%, and the miss index is 0.93%.

(3) The performance, power consumption, and COP of the maize metering device developed are compared and analyzed. The results reveal that the quality of feed index of the developed novel centrifugal precision metering device exceeds 93.00% at a ground speed of 12~18 km/h, meeting the requirements of high-speed operation. In addition, the actual power consumption of Types B and C is about 85.00% and 98.00% lower than that of Type A. The COP of Type C is about 86 times and 12 times that of Types A and B, respectively. This implies that a judicious combination of a reasonable annular cavity diameter and centrifugal diameter change effect can ameliorate the augmented energy efficiency and more effectively cater to clean production requirements.

## Data availability statement

The raw data supporting the conclusions of this article will be made available by the authors, without undue reservation.

## Author contributions

MZ: Conceptualization, Investigation, Writing – original draft. PZ: Investigation, Software, Writing – original draft. XG: Methodology, Writing – review & editing. QL: Conceptualization, Methodology, Resources, Writing – original draft, Writing – review & editing.

## References

[B1] Bai Y. Deng X. Jiang S. Zhao Z. Miao Y. (2019). Relationship between climate change and low-carbon agricultural production: A case study in Hebei Province, China. Ecol. Indic. 105, 438–447. doi: 10.1016/j.ecolind.2018.04.003

[B2] Cisternas I. Velasquez I. Caro A. Rodriguez A. (2020). Systematic literature review of implementations of precision agriculture. Comput. Electron. Agric. 176, 105626. doi: 10.1016/j.compag.2020.105626

[B3] Dong J. Zhang S. Zheng Z. Wu J. Huang Y. Gao X. (2024). Development of a novel perforated type precision metering device for efficient and cleaner production of maize. J. Cleaner Product. 443, 140928. doi: 10.1016/j.jclepro.2024.140928

[B4] Du X. Liu C. (2023). Design and testing of the filling-plate of inner-filling positive pressure high-speed seed-metering device for maize. Biosyst. Eng. 228, 1–17. doi: 10.1016/j.biosystemseng.2023.02.008

[B5] Gao X. Cui T. Zhou Z. Yu Y. Xu Y. Zhang D.. (2021). DEM study of particle motion in novel high-speed seed metering device. Adv. Powder Technol. 32, 1438–1449. doi: 10.1016/j.apt.2021.03.002

[B6] Gao X. Xie G. Li J. Shi G. Lai Q. Huang Y. (2023). Design and validation of a centrifugal variable-diameter pneumatic high-speed precision seed-metering device for maize. Biosyst. Eng. 227, 161–181. doi: 10.1016/j.biosystemseng.2023.02.004

[B7] Gao X. Xie G. Xu Y. Yu Y. Lai Q. (2022a). Application of a staggered symmetrical spiral groove wheel on a quantitative feeding device and investigation of particle motion characteristics based on DEM. Powder Technol. 407, 117650. doi: 10.1016/j.powtec.2022.117650

[B8] Gao X. Zhao P. Li J. Xu Y. Huang Y. Wang L. (2022b). Design and experiment of quantitative seed feeding wheel of air-assisted high-speed precision seed metering device. Agriculture 12, Article 11. doi: 10.3390/agriculture12111951

[B9] Gao X. Zhou Z. Xu Y. Yu Y. Su Y. Cui T. (2020). Numerical simulation of particle motion characteristics in quantitative seed feeding system. Powder Technol. 367, 643–658. doi: 10.1016/j.powtec.2020.04.021

[B10] Hill J. Goodkind A. Tessum C. Thakrar S. Tilman D. Polasky S.. (2019). Air-quality-related health damages of maize. Nat. Sustainab. 2, 397–403. doi: 10.1038/s41893-019-0261-y

[B11] Ji Y. Liu S. Li J. (2018). Experimental and numerical studies on dense-phase pneumatic conveying of spraying material in venturi. Powder Technol. 339, 419–433. doi: 10.1016/j.powtec.2018.08.031

[B12] Kabeel A. E. Elkelawy M. Bastawissi H. A. E. Elbanna A. M. (2019). An experimental and theoretical study on particles-in-air behavior characterization at different particles loading and turbulence modulation. Alexandria Eng. J. 58, 451–465. doi: 10.1016/j.aej.2019.04.002

[B13] Kadam G. S. Pawade R. S. (2024). Water vapor cutting fluid assisted productive machining of Inconel 718. Mat. Manufact. Processes 39, 98–109. doi: 10.1080/10426914.2023.2190389

[B14] Lei X. Hu H. Wu W. Liu H. Liu L. Yang W.. (2021a). Seed motion characteristics and seeding performance of a centralised seed metering system for rapeseed investigated by DEM simulation and bench testing. Biosyst. Eng. 203, 22–33. doi: 10.1016/j.biosystemseng.2020.12.017

[B15] Lei X. Hu H. Yang W. Liu L. Liao Q. Ren W. (2021b). Seeding performance of air-assisted centralized seed-metering device for rapeseed. Int. J. Agric. Biol. Eng. 14, 79–87. doi: 10.25165/j.ijabe.20211405.5349

[B16] Li J. Lai Q. Zhang H. Zhang Z. Zhao J. Wang T. (2021). Suction force on high-sphericity seeds in an air-suction seed-metering device. Biosyst. Eng. 211, 125–140. doi: 10.1016/j.biosystemseng.2021.08.031

[B17] Li C. Cui T. Zhang D. Yang L. He X. Li Z.. (2023a). Design and experiment of a centrifugal filling and cleaning high-speed precision seed metering device for maize. J. Cleaner Product. 426, 139083. doi: 10.1016/j.jclepro.2023.139083

[B18] Li H. Ling L. Wen C. Liu H. Wu G. An X.. (2023b). Structural optimization method of rice precision direct seed-metering device based on multi-index orthogonal experimental. Front. Plant Sci. 14, 1183624. doi: 10.3390/agriculture12081094 37484474 PMC10359481

[B19] Liu X. Niu Z. Li M. Hou M. Wei L. Zhang Y.. (2023). Design and experimental research on disc-type seeding device for single-bud sugarcane seeds. Int. J. Agric. Biol. Eng. 16, 115–124. doi: 10.25165/j.ijabe.20231602.6973

[B20] Ren S. Yi S. (2022). Control Mechanism and experimental study on electric drive seed metering device of air suction seeder. Tehnicki Vjesnik 29, 1254–1261. doi: 10.17559/TV-20210908053555

[B21] Shah W. U. H. Lu Y. T. Liu J. H. Rehman A. Yasmeen R. (2023). The impact of climate change and production technology heterogeneity on China’s agricultural total factor productivity and production efficiency. Sci. Total Environ. 907, 168027. doi: 10.1016/j.scitotenv.2023.168027 37898215

[B22] Sun J. Chen H. Duan J. Liu Z. Zhu Q. (2020). Mechanical properties of the grooved-wheel drilling particles under multivariate interaction influenced based on 3D printing and EDEM simulation. Comput. Electron. Agric. 172, 105329. doi: 10.1016/j.compag.2020.105329

[B23] Sun X. Niu L. Cai M. Liu Z. Wang Z. Wang J. (2023). Particle motion analysis and performance investigation of a fertilizer discharge device with helical staggered groove wheel. Comput. Electron. Agric. 213, 108241. doi: 10.1016/j.compag.2023.108241

[B24] Tang H. Xu F. Guan T. Xu C. Wang J. (2023). Design and test of a pneumatic type of high-speed maize precision seed metering device. Comput. Electron. Agric. 211, 107997. doi: 10.1016/j.compag.2023.107997

[B25] Tang H. Xu C. Wang Z. Wang Q. Wang J. (2022). Optimized design, monitoring system development and experiment for a long-belt finger-clip precision corn seed metering device. Front. Plant Sci. 13, 814747. doi: 10.3390/agriculture12081094 35154226 PMC8831804

[B26] Wang J. Qi X. Xu C. Wang Z. Jiang Y. Tang H. (2021). Design evaluation and performance analysis of the inside-filling air-assisted high-speed precision maize seed-metering device. Sustainability 13, 5483. doi: 10.3390/su13105483

[B27] Wang C. Yang H. He J. Kang K. Li H. (2023). The influence of seed variety and high seeding speed on pneumatic precision seed metering. Engenharia Agrícola 43, e20220183. doi: 10.1590/1809-4430-eng.agric.v43n3e20220183/2023

[B28] Wei H. H. Zhang K. P. Chai N. Wang Y. Li Y. L. Yang J.. (2023). Exploring low-carbon mulching strategies for maize and wheat on-farm: Spatial responses, factors and mitigation potential. Sci. Total Environ. 906, 167441. doi: 10.1016/j.scitotenv.2023.167441 37774862

[B29] Xiong L. Shah F. R. Wu W. (2022). Environmental and socio-economic performance of intensive farming systems with varying agricultural resource for maize production. Sci. Total Environ. 850, 158030. doi: 10.1016/j.scitotenv.2022.158030 35973532

[B30] Zhao P. Gao X. Su Y. Xu Y. Huang Y. (2024). Investigation of seeding performance of a novel high-speed precision seed metering device based on numerical simulation and high-speed camera. Comput. Electron. Agric. 217, 108563. doi: 10.1016/j.compag.2023.108563

[B31] Zhu Y. Zhang Y. Piao H. (2022). Does agricultural mechanization improve the green total factor productivity of China’s planting industry? Energies 15, 940. doi: 10.3390/en15030940

